# Defining specificity and on-target activity of BH3-mimetics using engineered B-ALL cell lines

**DOI:** 10.18632/oncotarget.7204

**Published:** 2016-02-05

**Authors:** Brian Koss, Jeremy Ryan, Amit Budhraja, Katherine Szarama, Xue Yang, Madhavi Bathina, Michael H. Cardone, Zaneta Nikolovska-Coleska, Anthony Letai, Joseph T. Opferman

**Affiliations:** ^1^ Department of Cell and Molecular Biology, St. Jude Children's Research Hospital, Memphis, TN, USA; ^2^ Department of Adult Oncology, The Dana-Farber Cancer Institute, Harvard Medical School, Boston, MA, USA; ^3^ Integrated Program in Biomedical Sciences, University of Tennessee Health Science Center, Memphis, TN, USA; ^4^ Eutropics Incorporated, Cambridge, MA, USA; ^5^ Department of Pathology, University of Michigan Medical School, Ann Arbor, MI, USA

**Keywords:** BH3 mimetics, apoptosis, BCL-2, cancer model, drug development

## Abstract

One of the hallmarks of cancer is a resistance to the induction of programmed cell death that is mediated by selection of cells with elevated expression of anti-apoptotic members of the BCL-2 family. To counter this resistance, new therapeutic agents known as BH3-mimetic small molecules are in development with the goal of antagonizing the function of anti-apoptotic molecules and promoting the induction of apoptosis. To facilitate the testing and modeling of BH3-mimetic agents, we have developed a powerful system for evaluation and screening of agents both in culture and in immune competent animal models by engineering mouse leukemic cells and re-programming them to be dependent on exogenously expressed human anti-apoptotic BCL-2 family members. Here we demonstrate that this panel of cell lines can determine the specificity of BH3-mimetics to individual anti-apoptotic BCL-2 family members (BCL-2, BCL-X_L_, BCL-W, BFL-1, and MCL-1), demonstrate whether cell death is due to the induction of apoptosis (BAX and BAK-dependent), and faithfully assess the efficacy of BH3-mimetic small molecules in pre-clinical mouse models. These cells represent a robust and valuable pre-clinical screening tool for validating the efficacy, selectivity, and on-target action of BH3-mimetic agents.

## INTRODUCTION

One of the hallmarks of cancer is the ability of cancer cells to overcome the induction of apoptosis that should be triggered when they violate normal cellular checkpoints. To do so, cancer cells commonly select for the elevated expression of anti-apoptotic proteins such as BCL-2, BCL-X_L_, BCL-W, BFL-1, or MCL-1 to antagonize the induction of cell death [1]. These anti-apoptotic BCL-2 molecules possess a hydrophobic binding pocket that is capable of binding the BH3-domain of pro-apoptotic BCL-2 family members [2]. It is this protein-protein interaction that allows BH3-only molecules (e.g. BIM, PUMA, NOXA, BID, BAD, etc.) to inhibit anti-apoptotic proteins and promote the death of cells by activating the pro-apoptotic effector molecules, BAX and BAK [3]. To counter the apoptotic resistance of cancer cells, researchers in academia and in the pharmaceutical industry have focused on the development of small molecular inhibitors of anti-apoptotic proteins, known as BH3-mimetic molecules [1]. By mimicking the activity of BH3-only molecules, small molecule BH3-mimetics can act to pharmacologically promote the release of pro-apoptotic BCL-2 family members from the anti-apoptotic proteins, thus pushing cancer cells to undergo apoptosis.

A number of strategies are used to test and evaluate BH3-mimetic drugs. In many cases, the screening for BH3-mimetic agents utilizes the displacement of recombinant anti-apoptotic proteins as assessed by fluorescent polarization or surface plasmon resonance [4, 5]. While these techniques are effective for high-throughput screening and evaluation of candidate small molecules, they are performed *in vitro* and do not assess biological processes including membrane permeability, specificity of interaction, and off-target effects that require cell based evaluation. As a secondary screen, it is common to test the efficacy of BH3-mimetics in a panel of cell lines. To this aim, researchers have used a variety of techniques including gene silencing by shRNA or BH3-profiling to identify cancer cell lines that are dependent on individual anti-apoptotic BCL-2 family members [6–9]. Therefore, the efficacy of a given BH3-mimetic in one of these cell lines is often evidence of the specificity of the BH3-mimetic. Unfortunately, often these cell lines represent a spectrum of different malignancies or sub-types making it challenging to compare the responses of one cell line with one another. Furthermore, these cells typically originate from human cancers requiring that *in vivo* pre-clinical testing be done in xenografts of immune compromised recipients. BH3 mimetics that are working “on pathway” should be dependent upon the expression of the multi-domain effectors BAX and BAK. However, human cancer cell lines are rarely deficient in both the pro-apoptotic effectors BAX and BAK; therefore, demonstration of on-target, pro-apoptotic activity of BH3-mimetics is challenging.

To aid in the development and testing of BH3-mimetic agents, we developed a panel of leukemia cell lines arising from a common parental population that have been engineered to be dependent on human anti-apoptotic BCL-2 family members. These mouse leukemia cells are suitable for cell-based screening as well as for testing in immune competent mouse models to allow the screening for toxic effects of the BH3-mimetics. By expressing human anti-apoptotic molecules, the transplanted leukemic cells can respond to treatment with small molecules designed for inhibition of human protein targets. Lastly, to demonstrate that the BH3-mimetics are acting in an “on-target” mechanism, we have generated cell lines that are deficient in their ability to undergo apoptosis by genetically ablating the multi-domain apoptotic effectors, *Bax* and *Bak*. Thus, this panel of re-programmed B-ALL cells represents an important tool to assess the specificity and potency of BH3-mimetic small molecules both in culture and *in vivo*.

## RESULTS

### Engineering BCR-ABL^+^ B-ALL cells to express human anti-apoptotics

**Figure 1:**
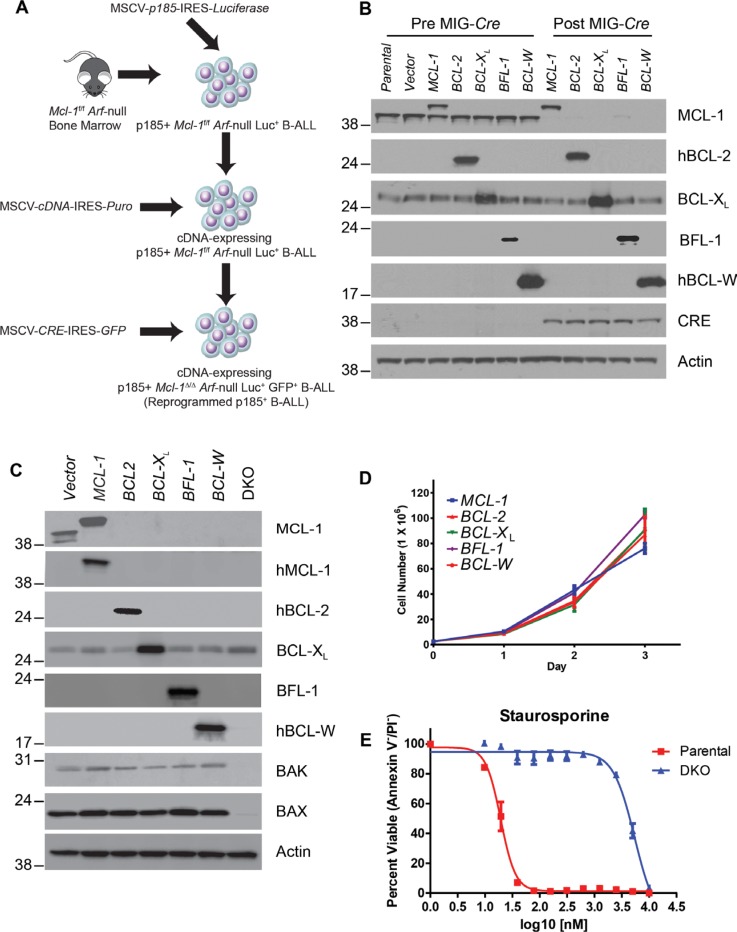
Re-programming of BCR-ABL^+^ B-ALL cell lines expressing human anti-apoptotic BCL-2 family members (**A**) Schematic illustrating the design of the re-programmed leukemia cell panel. Bone marrow from *Mcl-1*^f/f^ *Arf*^−/−^ mice was isolated, transduced with *p185*-IRES-*Luc2* (luciferase) and cultured without cytokines or stroma. Resulting transformed cells were transduced with cDNAs encoding human versions of anti-apoptotic molecules. Stably expressing cells were then transduced with *Cre*-*IRES*-*GFP* to induce the deletion of endogenous *MCL-1*. (**B**) Cell lysates from cells before and after CRE expression were resolved and immunoblotted for the expression of MCL-1, human BCL-2, BCL-X_L_, BFL-1, human BCL-W, and CRE. Actin serves as loading control and molecular weight markers (kD) are indicated. Leukemic cells in which *Mcl-1*-deletion is rescued by co-deletion of *Bax* and *Bak* are refered to as DKO cells. (**C**) Immunoblot of GFP^+^ sorted single-cell clones isolated by flow cytometry from B. Actin serves as loading control and molecular weight markers (kD) are indicated. (**D**) Clones of re-programmed B-ALL cells were cultured and viable cell number measured over time by trypan blue exclusion. Error bars reflect the standard error of the mean (SEM) of three independent experiments (*n* = 3). (**E**) Response of p185^+^ DKO B-ALL cells or wild-type p185^+^ B-ALL (Parental) to treatment with various concentrations of staurosporine as measured by Annexin-V^−^ and propidium iodide (PI)^−^ viable cells. Error bars indicate the SEM of three independent experiments (*n* = 3).

We have previously demonstrated that the anti-apoptotic activity of endogenous Mcl-1 is essential to maintain the survival of murine BCR-ABL-expressing B-lineage acute lymphoblastic leukemia (B-ALL) cell lines despite the concomitant expression of other anti-apoptotic molecules [10]. However, the ectopic expression of other anti-apoptotic BCL-2 family members can override the requirement for endogenous MCL-1 in these leukemic cells [10]. We sought to use this model to develop a panel of engineered, “re-programmed” B-ALL cell lines in which the endogenous *Mcl-1* was replaced by human versions of anti-apoptotic genes. To do so, *Mcl-1*^f/f^ *Arf*-deficient pre-B cells expressing the p185 isoform of BCR-ABL (hereafter referred to as p185^+^ B-ALL) were stably transduced with cDNAs encoding human BCL-2, BCL-X_L_, BCL-W, MCL-1, or BFL-1 (also known as BCL2A1). Following drug selection, these cells were further transduced with *Cre*-IRES-*GFP* to delete the endogenous *Mcl-1* (Figure 1A). The expression of human anti-apoptotic BCL-2 family members, but not an empty vector, was capable of supporting the outgrowth of p185^+^ B-ALL cells that had efficiently deleted endogenous *Mcl-1* from the cultures (Figure 1B). Single-cell clones were sorted based on GFP expression and tested by immunoblot to detect the loss of endogenous MCL-1 and exogenous BCL-2 family member expression (Figure 1C). These single cell clones were similar in their growth kinetics (Figure 1D).

Cells lacking both pro-apoptotic effector molecules BAX and BAK (referred to as DKO cells) are resistant to the induction of apoptosis [3, 11]. Thus, we sought to generate p185^+^ B-ALL cell lines defective in the core apoptotic pathway to use as controls to define whether tested BH3-mimetics are inducing leukemic death by triggering apoptosis. To do so, *Mcl-1* conditional *Arf*-deficient mice were bred to mice bearing conditional alleles of *Bax* (*Bax*^f/f^) on a *Bak*-deficient background. Bone marrow (BM) from these mice was harvested and transduced with the p185-IRES-*Cre* oncofusion virus to generate p185^+^ B-ALL cells in which *Mcl-1*-deletion is rescued by loss of both BAX and BAK (hereafter referred to as DKO p185^+^ B-ALL cells). Indeed, when p185^+^ B-ALL cells were compared with DKO p185^+^ B-ALL cells, the DKO p185^+^ B-ALL cells were markedly resistant to cell death induced by treatment with the pan-kinase inhibitor staurosporine (Figure 1E). Therefore, the DKO p185^+^ B-ALL cells are inherently resistant to the induction of apoptosis.

### Testing the anti-apoptotic dependence of the engineered B-ALL cell lines

**Figure 2:**
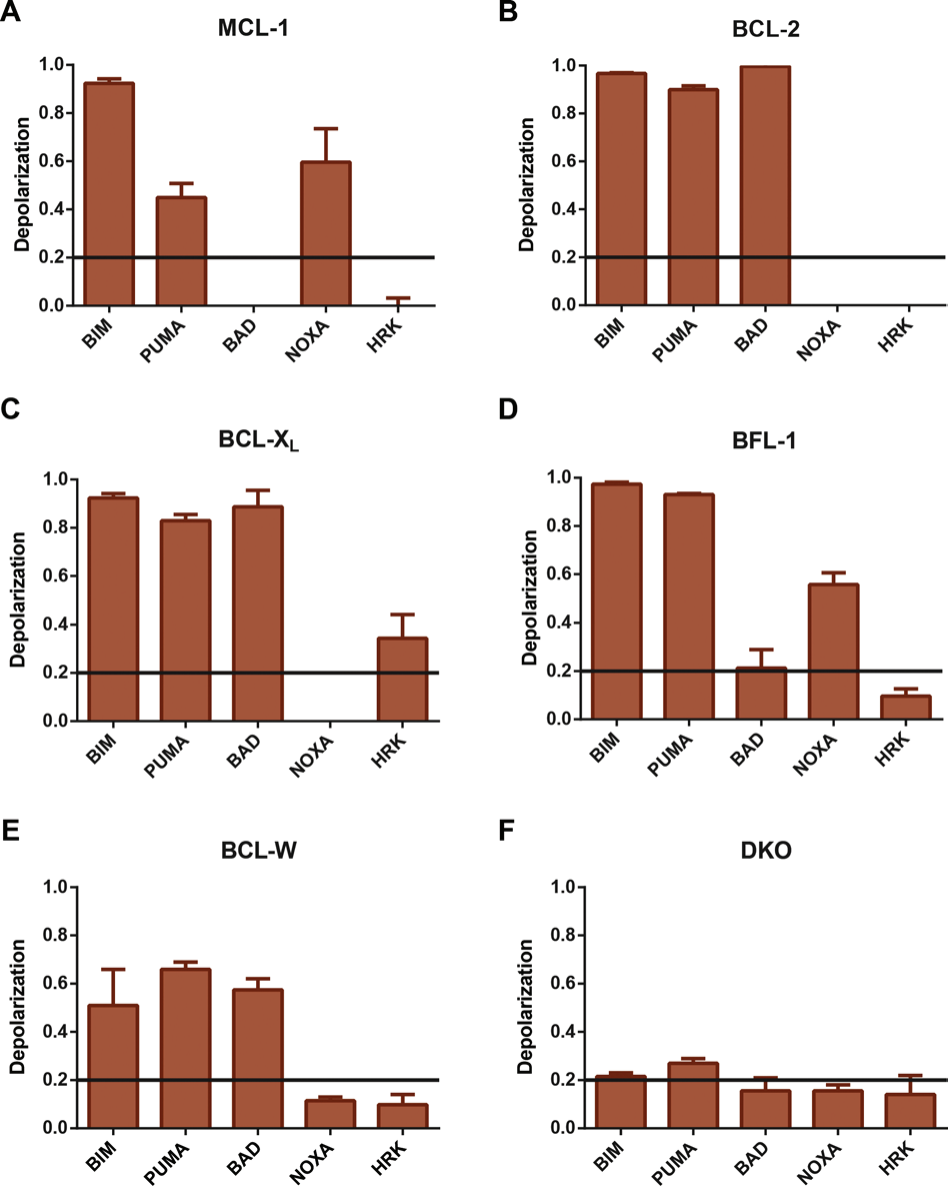
BH3-profiling for panel of re-programmed p185^+^ B-ALL cells Re-programmed p185^+^ B-ALL cell lines, in which endogenous MCL-1 was replaced by indicated human anti-apoptotic BCL-2 family members, were gently permeablized with digitonin and incubated with BH3-domain peptides derived from indicated BH3-only molecules (BIM, PUMA, BAD, or HRK at 10 μM or NOXA at 100 μM) and then labeled with JC-1, a marker of mitochondrial membrane potential. Cells were then analyzed by fluorescence spectroscopy to detect the fluorescent emission of JC-1. BIM and PUMA peptides should depolarize all cell lines expressing BAX and BAK. BAD peptide is specific for BCL-2, BCL-X_L_, and BCL-W; NOXA should depolarize cells expressing MCL-1 or BFL-1; HRK peptide is specific for BCL-X_L_, but not other anti-apoptotics. The average of *n* = 3 replicate fluorescent spectroscopy responses of (**A**) human *MCL-1* re-programmed cells, (**B**) human *BCL-2* re-programmed cells, (**C**) human *BCL-X*_L_ re-programmed cells, (**D**) *BFL-1* re-programmed cells, (**E**) human *BCL-W* re-programmed cells, and (**F**) DKO p185^+^ B-ALL cells are presented. The data presented are the average of 3 independent experiments (*n* = 3), all of which were performed in triplicate. Error bars denote standard of deviation.

To confirm the anti-apoptotic dependence of our engineered p185^+^ B-ALL cell lines, we used BH3-profiling. This technique takes advantage of the selective interactions between BH3-only proteins and individual anti-apoptotic BCL-2 family members and uses mitochondrial membrane depolarization as a readout [12]. Mildly permeablized cells are treated with peptides derived from BH3-only family members to determine their anti-apoptotic dependence as measure by depolarization of their mitochondrial membrane potential induced by mitochondrial outer membrane permeabilization (MOMP). The response to treatment with different peptides allows the assessment of anti-apoptotic dependence; BAD BH3-peptides are selective for BCL-2, BCL-X_L_, and BCL-W; NOXA BH3-peptides are selective for MCL-1 and BFL-1; HRK BH3-peptides are selective for BCL-X_L_; PUMA and BIM BH3-peptides work on any anti-apoptotic molecule [12, 13]. To assess the dependence of our re-programmed p185^+^ B-ALL cell lines, cells were digitonin-permeablized and treated with the panel of BH3-peptides and mitochondrial membrane potential measured by staining cells with the membrane potential dye JC-1, which fluoresces red in the matrix of healthy mitochondria and green in depolarized cells. As expected, both BIM and PUMA peptides depolarized all of the leukemic cells tested except for those lacking BAX and BAK, further illustrating that the DKO p185^+^ B-ALL cells are defective in the core apoptotic pathway (Figure 2F). In contrast, incubation of the p185^+^ B-ALL cells with the BAD peptide only induced the depolarization of cells in which exogenous human BCL-2, BCL-X_L_, and BCL-W replaced endogenous MCL-1 (Figure 2B, 2C, 2E). In contrast, NOXA peptide depolarized cells expressing exogenous MCL-1 and BFL-1, but not other anti-apoptotic molecules (Figure 2A and 2D). Lastly, treatment with the HRK peptide only depolarized the cells expressing exogenous human BCL-X_L_ (Figure 2C). As expected, DKO p185^+^ B-ALL leukemic cells were resistant to depolarization mediated by all BH3-only peptides, consistent with the requirement for multi-domain effector pro-apoptotic molecules for the induction of MOMP (Figure 2F). Therefore, BH3-profiling confirmed that re-programmed leukemic cells are dependent on the exogenously expressed human anti-apoptotic BCL-2 proteins.

### Testing BH3-mimetic agents on engineered B-ALL cell lines

To demonstrate the utility of using our re-programmed p185^+^ B-ALL cell lines for testing potential BH3-mimetics, we cultured this panel of cell lines with various doses of BH3-mimetic small molecules. The panel of re-programmed p185^+^ B-ALL cells (expressing exogenous BCL-2, BCL-X_L_, BCL-W, BFL-1, or MCL-1) or DKO leukemic cells lacking the intrinsic apoptotic pathway were cultured with titrated doses of BH3-mimetic agents and then analyzed for induction of apoptosis 24 hours later by staining with Annexin-V and propidium iodide (PI).

**Figure 3:**
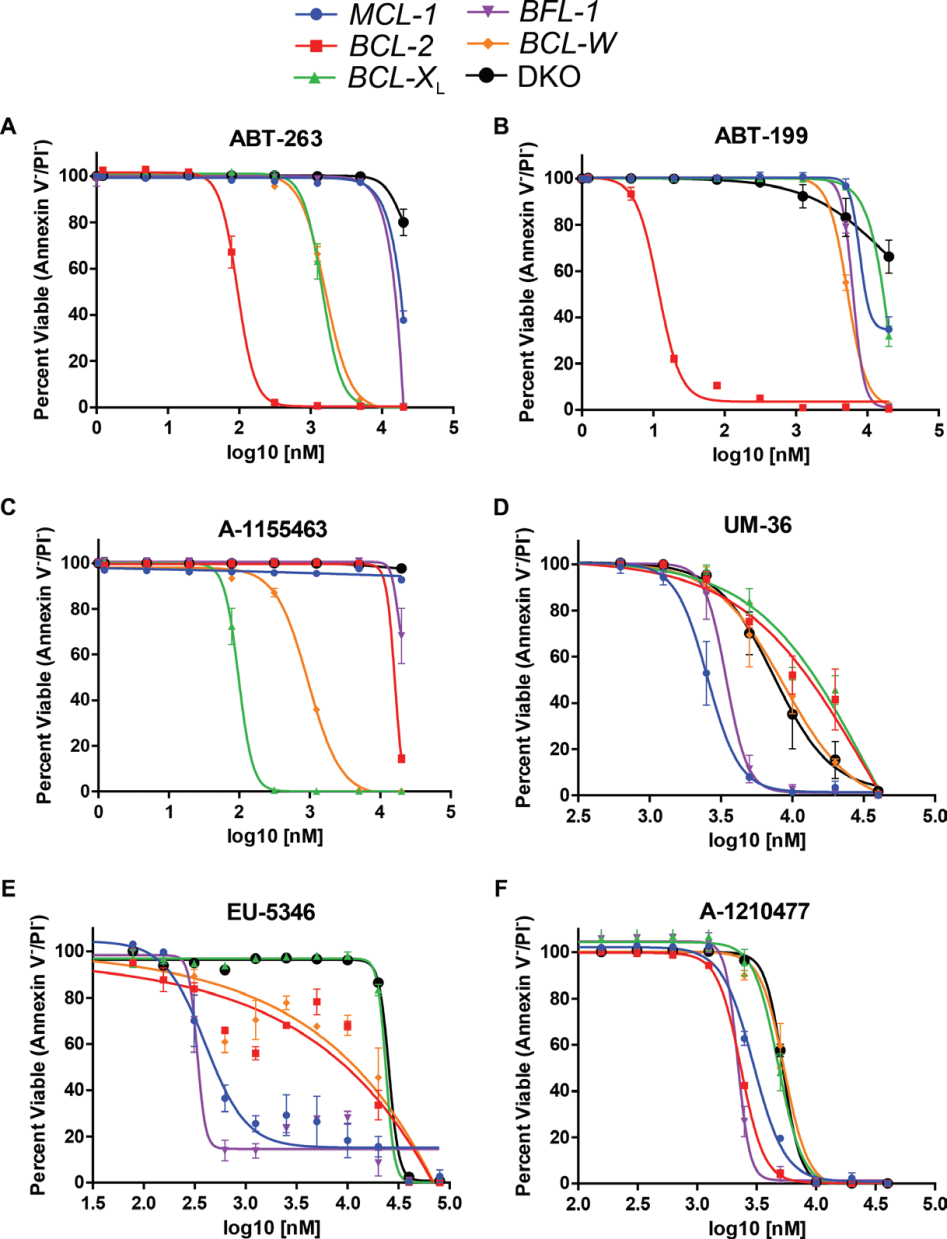
Response of re-programmed p185^+^ B-ALL cell lines to BH3-mimetic small molecules Re-programmed B-ALL cell lines were seeded and treated with indicated doses of BH3-mimetic small molecules. At 24 hours, the cell viability was analyzed by staining the cells with Annexin-V and propidium iodide (PI) and quantifying the viable cells. Data displayed are the average of 3 independent experiments (*n* = 3) and error bars indicate the SEM. The response of the cell lines to (**A**) navitoclax, ABT-263; (**B**) venetoclax, ABT-199; (**C**) AbbVie's BCL-XL inhibitor, A-1155463; (**D**) University of Michigan MCL-1 inhibitor, UM-36; (**E**) Eutropics Pharmaceuticals MCL-1 inhibitor, EU-5346; (**F**) AbbVie MCL-1 inhibitor, A-1210477 are presented. All drugs were solubilized in DMSO and to control for toxicity DMSO vehicle controls were performed on each cell line for each replicate. Cell death induced in DKO cells is considered to be non-apoptotic.

We investigated the response of the panel of p185^+^ B-ALL cell lines to several of the most advanced BH3-mimetic agents (ABT-263 and ABT-199) which are already being tested in clinical trials. ABT-263 (navitoclax) is a BH3-mimetic that is specific for BCL-2, BCL-X_L_, and BCL-W, but does not inhibit MCL-1 or BFL-1 [14]. ABT-263 has shown some promise in clinical trials, but is associated with the induction of thrombocytopenia due to the requirement for BCL-X_L_ in maintaining mature platelet survival [15–17]. ABT-199 (venetoclax) is a potent, specific BCL-2 inhibitor that does not inhibit other anti-apoptotic molecules and avoids the thrombocytopenia associated with inhibition of BCL-X_L_ [18]. Venetoclax has exhibited dramatic responses in several tumor types including chronic lymphocytic leukemia (CLL) treatment and is in advanced clinical trials [19–21]. Treatment of the panel of p185^+^ B-ALL cells with navitoclax induced the cell death only of leukemic cells re-programmed to be dependent on BCL-2 (IC_50_ = 94 nM), BCL-X_L_ (IC_50_ = 146 nM), and BCL-W (IC_50_ = 162 nM) (Figure 3A). In contrast, re-programmed p185^+^ B-ALL cells expressing MCL-1 or BFL-1 were extremely resistant to navitoclax, similar to the resistance of cells lacking the pro-apoptotic effectors BAX and BAK (Figure 3A). When the panel was treated with venetoclax, only p185^+^ B-ALL cells re-programmed with human BCL-2 (IC_50_ = 12 nM) responded by inducing apoptosis, while cells expressing other anti-apoptotic molecules were resistant to ABT-199-mediated killing (IC_50_ > 5 μM) (Figure 3B). Neither navitoclax nor venetoclax induced death in DKO p185^+^ B-ALL cells, except at high concentrations (IC_50_ > 20 μM), indicating that the main mechanism of action of these agents at effective concentrations in p185^+^ B-ALL cells is induction of intrinsic apoptosis (Figure 3A and 3B).

Despite the thrombocytopenia associated with BCL-X_L_ inhibition, there is desire to generate specific BCL-X_L_ inhibitors to aid treatment of BCL-X_L_-dependent malignancies. One such molecule, A-1155463 is a BH3-mimetic reported to specifically inhibit the activity of BCL-X_L_ [22–23]. When the re-programmed leukemic cells were treated with, A-1155463, the BCL-X_L_ expressing cells were very sensitive (IC_50_ = 97 nM) and BCL-W expressing cells were also killed at higher concentrations (IC_50_ = 950 nM) (Figure 3C). A-1155463 does not induce appreciable cell death in p185^+^ B-ALL cells re-programmed to be dependent on BFL-1, MCL-1, or BCL-2. Furthermore, A-1155463 appears to induce death by triggering intrinsic apoptosis as we were unable to detect death induced in DKO p185^+^ B-ALL cells (Figure 3C).

Elevated MCL-1 expression has been demonstrated to be an important mediator of resistance to responses to navitoclax and venetoclax [24–27]. Therefore, there is intense interest in the development of MCL-1-selective inhibiting BH3-mimetics. We evaluated three MCL-1 inhibitors that have been recently reported in the literature (UM-36, EU-5346, and A-1210477). A UM-36 is a BH3-mimetic small molecule that was reported to kill cell lines only when those cells express MCL-1 [28, 29]. Another MCL-1 selective BH3-mimetic, EU-5346, from Eutropics Pharmaceuticals has been reported to induce specific killing of MCL-1-dependent cell lines [30]. A-1210477 is a tool MCL-1 inhibitor developed by AbbVie that has also been reported to induce death selectively in MCL-1-dependent cell lines [31–33]. When UM-36 was applied to the panel of re-programmed leukemia cells it induced apoptosis in p185^+^ B-ALL cells re-programmed to express human MCL-1 (IC_50_ = 2.5 μM) and, to a lesser extent, in cells expressing BFL-1 (IC_50_ = 3.4 μM) (Figure 3D). In contrast, the p185^+^ B-ALL cells expressing other anti-apoptotic BCL-2 family members were as resistant as DKO p185^+^ B-ALL cells (IC_50_ > 6.8 μM). These data indicate that while UM-36 may have some specificity, the potency of the molecule is very meager across all cell types. When EU-5346 was tested against the panel of re-programmed p185^+^ B-ALL cell lines, it was most effective at inducing killing of the BFL-1 expressing cell lines (IC_50_ = 334 nM) and MCL-1 expressing cells (IC_50_ = 403 nM) (Figure 3E). In contrast, EU-5346 was not potent against cells expressing other anti-apoptotic molecules or in cells lacking BAX and BAK (IC_50_ > 23 μM). These data indicate that EU-5346 exhibits some specificity to induce the death of p185^+^ B-ALL cells expressing BFL-1 or MCL-1. Despite the reports of efficacy of A-1210477 as a single agent in other cell types, it did not induce any selective cell death in any of our re-programmed p185^+^ B-ALL cells with death of BCL-2, BFL-1, and MCL-1 expressing cells only occurring at high concentrations (IC_50_ > 2 μM) (Figure 3F). At marginally higher concentrations (IC_50_ = 5.3 μM) A-1210477 also induced the death of DKO cells suggesting that these concentrations it induces cell death in a BAX and BAK-independent manner.

### Evaluating the *in vivo* response of re-programmed leukemic cell to BH3-mimetic drugs

**Figure 4:**
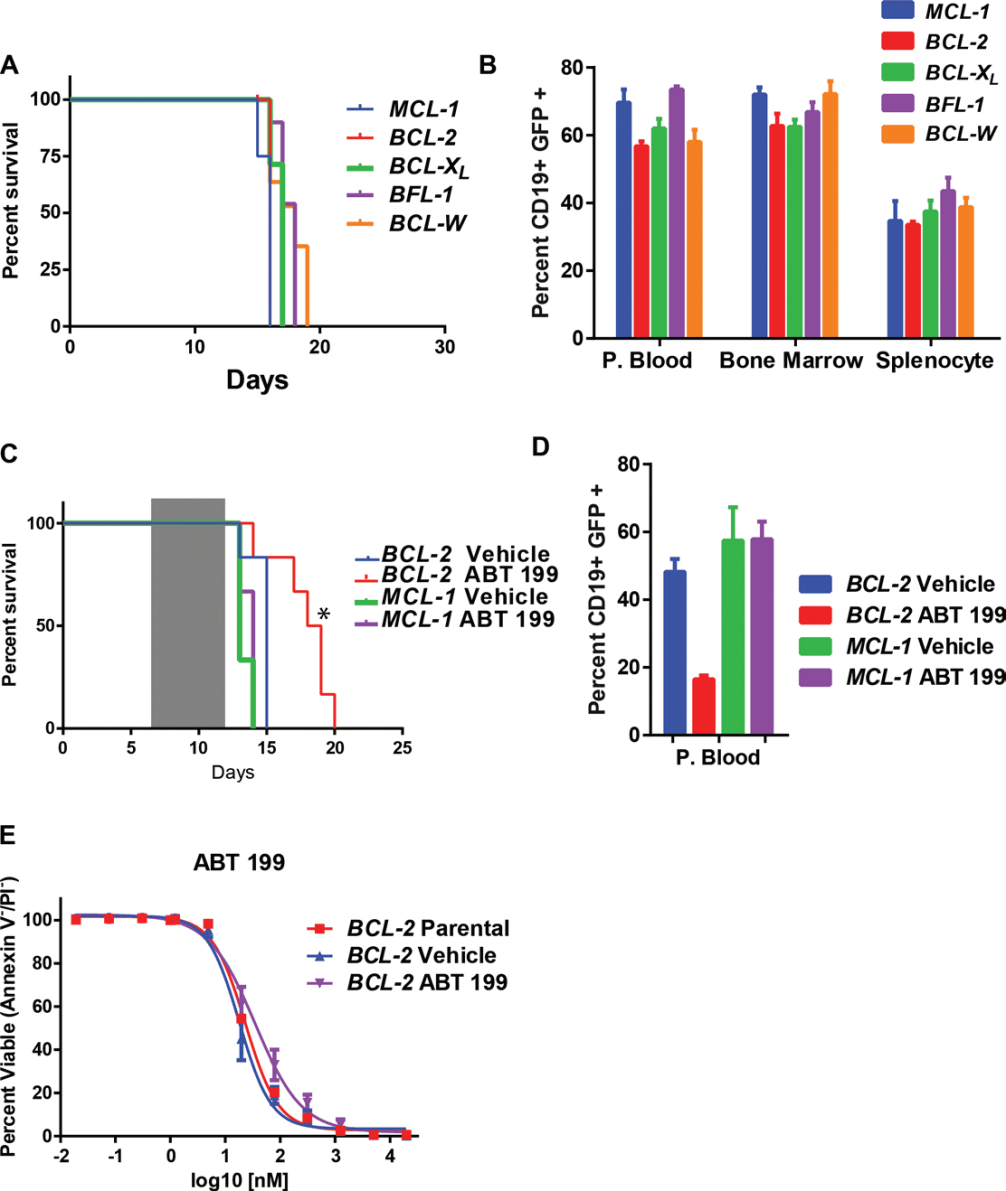
Testing re-programmed p185+ B-ALL cells response in immune competent recipients (**A**) Kaplan-Meier survival curve of recipient mice receiving re-programmed B-ALL cell lines (1 × 10^5^ cells per recipient). Mice were sacrificed when they displayed signs of frank leukemia. Each cohort contained 5 mice and log-rank test was performed and indicated no statistical significance between mice bearing any of the cell lines. (**B**) Analyses of peripheral blood (P. Blood), bone marrow, and splenocytes for the percentages of leukemic (GFP^+^) cells present. Each bar represents the average GFP^+^ percentage of an individual re-programmed B-ALL cell line expressing indicated anti-apoptotic family member in each tissue. Error bars indicate the standard error of the mean of *n* = 5 mice per group. No statistically significant differences were detected between individual anti-apoptotic BCL-2 family members. (**C**) Kaplan-Meier survival curve of recipient mice receiving either MCL-1 or BCL-2 re-programmed p185^+^ cell lines (1 × 10^5^ cells per recipient). Five days after transplant the mice were treated daily with either vehicle or ABT-199 by oral gavage for 5 days (treatment time shown in gray box). Mouse condition was monitored daily and sacrificed when moribund. Asterisk (*) denotes *p* < 0.001 by Log Rank Test. (**D**) Flow cytometric analyses of peripheral blood from treated mice (as described in panel C) on day 13. Each bar represents the average and standard error of the mean of at least 5 mice per group. Peripheral blood was stained with CD19 and the GFP expression in the transplanted leukemic cells. (**E**) Human BCL-2 expressing p185^+^ B-ALL cells were isolated from moribund mice (as described in C) either treated *in vivo* with ABT-199 or with vehicle and expanded *in vitro*. As a comparison, the parental BCL-2 re-programmed p185^+^ B-ALL cells which were never injected into recipients were also treated. The cells were treated with ABT-199 in culture at indicated doses. At 24 hours, the cell viability was analyzed by staining the cells with Annexin-V and propidium iodide (PI) and quantifying the viable cells. Data displayed are the average of 3 independent experiments (*n* = 3) and error bars indicate the SEM.

One of the strengths of the p185^+^ B-ALL model system is the ability to transplant these leukemic cells into immune competent C57BL/6 recipient mice and give rise to a rapidly fatal leukemia [34–35]. Therefore, we sought to test whether the panel of re-programmed p185^+^ B-ALL cells could respond appropriately to BH3-mimetic treatment in immune competent recipients as a proof of principle. To this aim, we intravenously injected C57BL/6 mice with 1 × 10^5^ re-programmed p185^+^ B-ALL cells engineered to express green fluorescent protein (GFP^+^) and monitored the mice for leukemia progression. Irrespective of the expression of anti-apoptotic BCL-2 family members, the re-programmed p185^+^ B-ALL cells in which endogenous MCL-1 was replaced by human BCL-2, BCL-X_L_, BCL-W, MCL-1, or BFL-1 all succumbed to a fatal leukemia with a similar kinetic (Figure 4A). Furthermore, analyses of the peripheral blood, bone marrow, and spleens of the recipient mice all revealed similar percentages of leukemia as detected by flow cytometry for GFP expression (Figure 4B). Therefore, the re-programmed panel of leukemic cells exhibit similar abilities to give rise to a fatal leukemia.

To demonstrate the effectiveness of using our re-programmed leukemic cell lines to validate the efficacy of BH3-mimetic agents *in vivo*, we transplanted 1 × 10^5^ p185^+^ B-ALL cells re-programmed to express either human BCL-2 or human MCL-1 into C57BL/6 recipients. The leukemic cells express GFP and luciferase to facilitate their detection in the peripheral blood of recipient mice. On day 7 after transplant, recipient mice were treated either with ABT-199 (100 mg/kg) or vehicle control for 5 days at which point the treatment ceased and mice were continually monitored for leukemia progression as previously described [19]. Within 13 days, the recipient mice treated with vehicle control rapidly succumbed to leukemia irrespective of whether they were transplanted with p185^+^ B-ALL cells re-programmed with human BCL-2 or human MCL-1 (Figure 4C). In contrast, 5 days of treatment with venetoclax significantly extended the survival of recipient mice transplanted with re-programmed human BCL-2 expressing leukemic cells, but did not prolong the survival of MCL-1 expressing leukemic cells (Figure 4C). Peripheral blood analyses on day 13 demonstrate that only mice transplanted with human BCL-2 re-programmed leukemic cells responded to venetoclax treatment by decreasing the leukemic burden (Figure 4D). These data indicate that our engineered cell lines are capable of reflecting the *in vitro* sensitivity even when transplanted *in vivo* to C57BL/6 recipients.

Despite the survival delay, even mice bearing re-programmed BCL-2-dependent p185^+^ B-ALL cells that were treated with venetoclax for a 5 day window eventually developed a fatal leukemia. To address whether these cells had developed resistance to venetoclax *in vivo* or whether the cells survived the venetoclax treatment but remained sensitive, we harvested the leukemic cells from both venetoclax and vehicle treated mice at the time of sacrifice. The *ex vivo* leukemic cells were cultured in the absence of growth factors or supporting stroma. To test whether these cells retained sensitivity to venetoclax, the leukemic cells isolated from recipients (both vehicle and ventoclax-treated) were treated in culture with venetoclax and compared them to the parental human BCL-2 p185^+^ B-ALL re-programmed cells. No difference in the sensitivity of the human BCL-2 re-programmed leukemic cells to venetoclax was detected when we compared the parental human BCL-2 p185^+^ B-ALL cells to those that were isolated from the venetoclax-treated recipients (Figure 4E). These data indicate that the cells that survived the 5 days of *in vivo* treatment with venetoclax did not select for intrinsic cellular resistance (i.e. venetoclax insensitive), but maintained their dependence on the exogenous human BCL-2. It is possible that 5 days of treatment was insufficient to kill the leukemic cells *in vivo*, either due to insufficient drug exposure or the leukemia homing to niches that provided extrinsic survival signals such as cytokines [34]. Therefore when the treatment was ended, the leukemic cells that failed to die in response to venetoclax treatment emerged. These data would suggest that longer treatment with venetoclax, even as a single agent might be even more effective at delaying leukemia progression of the BCL-2-dependent leukemia.

## DISCUSSION

Here we report the development of a panel of engineered mouse p185^+^ B-ALL cells that have been re-programmed to be dependent on individual anti-apoptotic BCL-2 family members. These leukemic cells, which originate from a common precursor, are completely dependent on the exogenously expressed human anti-apoptotic proteins as assessed by BH3-profiling. The re-programmed leukemic cells faithfully recapitulate the response to the most advanced BH3-mimetic small molecules that are being used in the clinic. For example, only BCL-2, BCL-X_L_, or BCL-W re-programmed cells were killed by navitoclax, while venetoclax was remarkably potent in killing only BCL-2 re-programmed leukemic cells.

Our re-programmed B-ALL cells confirmed that A-1155463, a BCL-X_L_ inhibitor from AbbVie, also induces specific apoptosis of BCL-X_L_-expressing cells and to a lesser extent has potency in our BCL-Wre-programmed B-ALL cells. These data would predict that A-1155463 should be effective in treating BCL-X_L_-dependent leukemia. Indeed, A-1155463 has shown some promise in xenograft models, but it is hampered by its poor solubility and dose limiting toxicity of the vehicle [22]. We anticipate that further development will improve pharmacokinetics of this class of inhibitor, permitting dosages higher than 5 milligrams per kilogram in mouse models and oral delivery.

Our testing of MCL-1 inhibitors has unfortunately not revealed any of the tested BH3-mimetics to exhibit highly potent cell death selectively in MCL-1-dependent cells. UM-36 appears to have modest selectivity for MCL-1, with similar response in BFL-1-dependent leukemia, which correlates with the *in vitro* binding affinity of this class of MCL-1 inhibitor showing less selective inhibition of BFL-1 [29]. However, UM-36 also induces apoptosis in leukemic cells lacking BAX and BAK expression at higher concentrations, reflecting a narrow window of efficacy. Our testing of EU-5346 indicates that this inhibitor has modest potency, needing greater than 334 nM concentrations of the compound to elicit a response in cultured cells. Furthermore, re-programmed p185^+^ B-ALL cells expressing BFL-1 appear to be the most sensitive to EU-5346, with MCL-1 expressing cells responding at higher concentrations. Our data confirm that EU-5346 does not induce any cell death in BCL-X_L_-dependent cell lines, consistent with the fact that it was identified using a positive screen for MCL-1-specific inhibition and a counter screen against BCL-X_L_ inhibition [30]. At this time, it is not possible to treat mice with EU-5346 as it has high serum binding capacity (evident even *in vitro* assays that require 1% serum) and is poorly soluble; however, we look forward to testing improved versions of this potentially promising small molecule.

It was reported that A-1210477 is a specific inhibitor of MCL-1 and induces cell death in MCL-1-dependent human multiple myeloma cancer cells [32, 33]. However, we were unable to detect any specific induction of cell death in our re-programmed p185^+^ mouse B-ALL cell panel when treated with A-1210477. Several possibilities for the discrepancy exist. First, in the re-programmed p185^+^ B-ALL cells there may still be endogenous buffering capacity of endogenous anti-apoptotic BCL-2 family members. Therefore, the activity of A-1210477 may be insufficient to overcome this capacity due to its high *in vitro* specificity for MCL-1 [31, 33]. This interpretation would fit with the observation that A-1210477 efficacy can be potentiated by combination with navitoclax [32]. Secondly, it is possible that A-1210477 works more efficiently in human cancer cells than it does in mice. While our re-programmed mouse B-ALL cells do express human MCL-1, all of the other molecules in the cells are murine including other anti- and pro-apoptotic proteins such as NOXA, which is quite different in mouse and human [36]. Therefore, A-1210477 and other BH3-mimetics may trigger different responses in human versus mouse cells representing a potential caveat of this system. Lastly, it is possible that different cell types respond differently to A-1210477. Further work, including testing new “next-generation” MCL-1 inhibitors, should be able to clarify this question.

Interestingly, all three of the inhibitors designed to inhibit MCL-1 also appeared to have some degree of specificity for BFL-1-dependent cell lines. The reason for this is still unclear, but several possibilities exist. First, MCL-1 and BFL-1 have the most similar BH3-binding pockets of anti-apoptotic proteins; making is possible that inhibitors targeted against MCL-1 may have cross reactivity for BFL-1 [37]. Secondly, both BFL-1 and MCL-1 are short half-lived proteins; therefore it is possible that the BH3-mimetics may affect the protein stability similarly repressing MCL-1 and BFL-1 proteins [38–40]. Further studies will be necessary to identify why both MCL-1 and BFL-1 anti-apoptotic molecules appear to respond similarly to MCL-1 inhibiting BH3-mimetics and whether this will be a common feature of MCL-1 BH3-mimetics.

Successful screening of BH3-mimetic molecules depends upon the fidelity of the response of cell lines both in culture and in animal models. In culture, the re-programmed p185^+^ B-ALL cell lines faithfully recapitulate the specificity and potency of advanced BH3-mimetic small molecules including venetoclax and navitoclax. Therefore, we posit that our panel of re-programmed cells is ideal for the testing of next generation BH3-mimetics and is capable of determining potency, selectivity, and on-target induction of apoptosis. Furthermore, our panel of re-programmed cells can rapidly test the efficacy of BH3-mimetic molecules to induce apoptosis *in vivo*. The evaluation of leukemic response *in vivo* is important as endogenous growth factors are important mediators of resistance to response to therapy. An important strength of our system is that the cells can be frozen, thawed and injected into recipient mice without the need for lethal irradiation. This allows the cells to be banked and expanded for a cohort of mice which facilitates replicates and reproducibility. Another benefit of this system is that the recipient mice still have an endogenous immune system and thus any BH3-mimetic mediated effects on normal T and B lymphocytes can be evaluated. The re-programmed leukemic cells are marked with luciferase as well as GFP and therefore recipients can be monitored for survival by assessing endogenous luciferase luminescence using Xenogen imagery or by assessing peripheral blood for GFP expression. All of the re-programming has been done with human cDNAs encoding anti-apoptotic BCL-2 family members, allowing testing of inhibition on human and not just mouse anti-apoptotic molecules. This system allows evaluation of on-target activity and can demonstrate whether the cell death induced by BH3-mimetic small molecules is mediated by the induction of intrinsic apoptosis as we can test leukemic cells that are BAX and BAK-deficient. While this panel of re-programmed mouse B-ALL cells is clearly not a substitute for testing in human cancer cells which express different amounts of anti-apoptotic molecules, the panel has significant strengths that make it an excellent way to screen, validate, and test new BH3-mimetic inhibitors.

## MATERIALS AND METHODS

### Mice

*Mcl-1*-conditional; *Bax*-conditional Bak^−/−^; *Arf*^−/−^ mice have been described previously [41–43]. Recipients for leukemia are C57BL/6 mice obtained from the Jackson Laboratories. All mice were bred and utilized in accordance with St. Jude Children's Research Hospital (SJCRH) animal care and use committee.

### Plasmids, expression constructs, and generation of mutants

Human *BCL-X_L_*, *BCL-W*, and *BCL-2* cDNAs were from Dr. D. Green (St. Jude Children's Research Hospital, TN). *BFL-1* cDNA was kindly provided by Dr. C. Gélinas (Center for Advanced Biotechnology and Medicine, NJ). *BCR-ABL* (p185) oncofusion plasmid was from Dr. O. Witte (University of California Los Angeles, CA). Stable cells were generated by retroviral transduction and puromycin selection (Sigma Aldrich, MO, 2 μg per ml).

### Ecotropic retroviral production and cell transduction

Retroviruses were produced by transient transfection as previously described [44].

### Treatment of leukemia in recipient mice

Leukemic cells (1 × 10^5^ cells per recipient) were injected into sub-lethally irradiated (5 Gy) C57BL/6 recipients (Jackson Laboratory, ME) by tail-vein intravenous route. Peripheral blood was monitored for leukemia and recipients were observed for morbidity. Seven days after the transfer of leukemic cells, mice were treated with venetoclax (ABT-199) delivered by oral gavage. Venetoclax was formulated for oral delivery (gavage) in a mixture of 60% Phosal 50 PG, 30% PEG 400, and 10% EtOH and dosed at 100 mg/kg/day as previously described [19]. Treatment was given daily for 5 days (days 7–12) after which the mice were monitored daily for signs of leukemia progression.

### Cells and cell culture

Mouse p185^+^ *Arf*^−/−^ B-ALL were grown in RPMI (Life Technologies, CA) with 10% fetal bovine serum, 55 μM 2-mercaptoethanol, 2 mM glutamine, penicillin, and streptomycin. Navitoclax (ABT-263), venetoclax (ABT-199), A-1155463, and A-1210477 were kindly provided by AbbVie, IL. EU-5346 (also known as Compound #9) was provided by Eutropic Pharmaceutical, MA [30]. UM-36 was kindly provided by Dr. Nikolovska-Coleska [28, 29]. All compounds were solubilized in DMSO and added at indicated concentrations for cell culture assays.

### Western blotting and antibodies

Assessment of protein expression from cell lysates was performed as previously described [45]. Antibodies used were: anti-MCL-1 (Rockland Immunochemical, PA), anti-human MCL-1 (Cell Signaling, MA), anti-BCL-X_L_ (BD Biosciences, CA), anti-BCL-2 (Clone 6C8), total ABL antisera (Cell Signaling), anti-Cre (EMD Chemical, MA), anti-BCL-W (Cell Signaling), and anti-BFL-1 (Cell Signaling), anti-BAX (Cell Signaling), anti-BAK (Millipore), and anti-Actin (Millipore, MA). Secondaries were anti-rabbit or anti-mouse horseradish peroxidase-conjugated (Jackson Immunochemical, ME).

### Cell death experiments

BCR-ABL p185^+^ B-ALL cell lines re-programmed with human anti-apoptotic BCL-2 family members were seeded in 96-well round bottom plates (6 × 10^4^ cells/well). BH3-mimetic small molecules, solubilized in DMSO or DMSO vehicle controls were added at indicated concentrations and cells were in complete RPMI with 10% fetal calf serum (for ABT-263, ABT-199, and A-1155463) or 1% fetal calf serum (for UM-36, EU-5346, and A-1210477) as 10% serum significantly decreased the drug potency *in vitro* (likely due to serum binding). After 24 hours, the plates were centrifuged and cell viability was determined by staining with Annexin-V-APC and propidium iodide (BD Biosciences) and measured by flow cytometry using the high throughput sampler (HTS) on a FACSCanto II (BD Biosciences, CA).

### BH3 profiling

Compounds are from Sigma Aldrich, MO unless otherwise indicated. BH3 Peptides (New England Peptide, MA) were diluted to 2X their final concentrations in MEB (150 mM mannitol 10 mM HEPES pH 7.5, 50 mM KCl, 20 μM EDTA, 20 μM EGTA, 5 mM potassium succinate, 0.1% protease-free BSA (Gemini Bio-Products, CA) containing 10 mM 2-mercaptoethanol, 4 μM JC-1 (Enzo Life Science, NY), 20 μg/mL oligomycin (Enzo Life Sciences, NY), and 50 μg/mL digitonin. Fluotrac 200 384 well plates (Greiner Bio One, NC) were loaded with 15 μL per well of peptide/profiling solution in batches and frozen at −80°C. Peptides were used at final concentration of 10 μM for all BH3 proteins, except for NOXA (peptide derived from A BH3 domain of human NOXA) which was used at 100 μM [13]. Prior to profiling, a plate was thawed at 28°C. Cells were spun down at 500 xg for 5 min and suspended in MEB at a density of 1.33 × 10^6^ per mL. 15 μL of cell suspension was added to each well of the profiling plate, 20000 cells per well, and mitochondrial potential was monitored for three hours at five minute intervals on a Safire2 plate reader (Tecan, Switzerland) using Ex 545+/− 10 nm and Em 590+/− 10 nm to monitor only the potential sensitive, red fluorescent species of JC-1. Depolarization was determined by normalizing the area under each curve to the area of the fully depolarized 10 μM CCCP well or the fully charged inert control peptide PUMA calculated at Depolarization = 1-((Sample – CCCP)/(PUMA-CCCP)) [13].
